# Arms Race between Enveloped Viruses and the Host ERAD Machinery

**DOI:** 10.3390/v8090255

**Published:** 2016-09-19

**Authors:** Dylan A. Frabutt, Yong-Hui Zheng

**Affiliations:** Department of Microbiology and Molecular Genetics, Michigan State University, East Lansing, MI 48824, USA; frabuttd@msu.edu

**Keywords:** enveloped viruses, viral glycoproteins, endoplasmic reticulum-associated degradation, ERAD, unfolded protein response, UPR, ER stress

## Abstract

Enveloped viruses represent a significant category of pathogens that cause serious diseases in animals. These viruses express envelope glycoproteins that are singularly important during the infection of host cells by mediating fusion between the viral envelope and host cell membranes. Despite low homology at protein levels, three classes of viral fusion proteins have, as of yet, been identified based on structural similarities. Their incorporation into viral particles is dependent upon their proper sub-cellular localization after being expressed and folded properly in the endoplasmic reticulum (ER). However, viral protein expression can cause stress in the ER, and host cells respond to alleviate the ER stress in the form of the unfolded protein response (UPR); the effects of which have been observed to potentiate or inhibit viral infection. One important arm of UPR is to elevate the capacity of the ER-associated protein degradation (ERAD) pathway, which is comprised of host quality control machinery that ensures proper protein folding. In this review, we provide relevant details regarding viral envelope glycoproteins, UPR, ERAD, and their interactions in host cells.

## 1. Enveloped Viruses

Despite their vast diversity, animal viruses can be simply divided into two categories: non-enveloped viruses and enveloped viruses [1]. While non-enveloped viruses are wrapped with naked shells made of viral capsid proteins, enveloped viruses are covered with a lipid-bilayer, which is called a viral envelope. The viral envelope is obtained from progenitor host cells during the budding process, which can be a portion of plasma membrane or intracellular membrane. On the surface of the enveloped viruses, there are peplomers that project from the viral envelope, and play a critical role in viral infection. These peplomers are also described as spikes, which are made of viral envelope glycoproteins. Envelope spikes serve to identify and bind to viral receptors on the host cell surface, allowing viral entry into cells and the initiation of infection by mediating virus-cell fusion. Thus, the infectivity of enveloped viruses is absolutely dependent on the integrity of the viral envelope, and the functionality of the viral glycoproteins found therein.

Enveloped viruses are more stable than non-enveloped viruses under physiological conditions, at the expense of their sensitivity to high-temperature, low-pH, desiccation, or detergent-treatment, which limits their ability to withstand severe environments [2]. The entry of enveloped viruses requires the formation of a fusion pore between the viral envelope and the cell membrane, through which the viral genome is released into the cell. This fusion process is triggered by interactions between viral glycoproteins on the viral envelope and viral receptors on the cell surface, which can occur directly at the plasma membrane at neutral pH or in endocytic compartments at either low or neutral pH [3]. In addition, enveloped viruses can also enter cells through direct cell-to-cell contacts via virological synapses to provide a means by which the virus can cross the biophysical and immunological obstacles to infection [4]. The membrane penetration mechanism differs fundamentally in non-enveloped viruses, but similar strategies are used for their entry [5]. In general, enveloped animal viruses possess greater adaptability than non-enveloped animal viruses, and consequently, cause a number of severe diseases, such as acquired immunodeficiency syndrome (AIDS), influenza, severe acute respiratory syndrome (SARS), hemorrhagic fever, hepatitis, encephalitis, and microcephaly.

## 2. Virus Envelope Glycoproteins

The fusion between viral envelope and cell membranes is absolutely critical for the entry of enveloped viruses, which is usually triggered by the insertion of a viral envelope glycoprotein’s (Env) fusion peptide into the host membrane. The vast majority of viral fusion proteins are type I transmembrane proteins, which have a single transmembrane domain (TMD), with their *N*-terminus outside cells and *C*-terminus inside cells. Most viral envelope proteins have been modeled as existing in a less stable pre-fusion state or a stable post-fusion state. Many of these proteins also oligomerize into trimeric fusogenic complexes in their post-fusion states, forming trimeric hairpin structures on the viral envelope.

Based on their structural and mechanistic properties, viral fusion proteins have been classified into three distinct classes [6]. Class I fusion proteins are found in influenza viruses, paramyxoviruses, retroviruses, and filoviruses. These envelope proteins are first expressed as a polypeptide precursor and then cleaved by cellular proteases, yielding a transmembrane protein with an amino-terminal fusion peptide and a surface protein, which are attached either non-covalently or by a disulfide-bond. The core of the class I protein fusogenic domain is predominantly composed of α-helices, which contain an *N*-terminally located fusion peptide. These proteins trimerize and form a central coiled-coil structure with a three-α-helix bundle in the pre-fusion state, which refolds into a six-α-helix bundle in the post-fusion state.

Class II fusion proteins are found in flaviviruses, hepaciviruses, alphaviruses, togaviruses, and Rift Valley fever viruses. They depend on a viral chaperone for folding, which is produced from the same polypeptide precursor where they are arrayed in tandem. When the chaperone is cleaved off, the fusion protein gains the fusogenic activity. These fusion proteins are mostly made of β-sheets and exist as homo- or hetero-dimers with the fusion peptides buried in internal loops in the pre-fusion state. In the post-fusion state, these proteins undergo self-rearrangement into stable trimeric hairpins, exposing the fusion peptide and resulting in viral and host membrane fusion.

Class III fusion proteins are found in rhabdoviruses, herpesviruses, and baculoviruses. These proteins are directly translated as a single protein from viral mRNA without protease cleavage, and trimerize in both pre- and post-fusion states. Notably, they combine structural signatures found in both classes I and II, which include a central trimeric coiled coil, three domains predominantly made of β-sheets, and internal fusion peptides in the pre-fusion state. However, unlike in class I and II fusion proteins, the pre-fusion and post-fusion states are reversible in class III fusion proteins.

In addition to these structural features, viral fusion proteins are subject to *N*-glycosylation at varying degrees. Although some viral envelope proteins such as the dengue virus (DENV) E protein are glycosylated at relatively low levels [7], most of the other important human viruses are subject to heavy glycosylation. For example, human immunodeficiency virus type 1 (HIV-1) Env precursor gp160 has ~34 potential *N*-linked glycosylation sites (PNGSs): ~30 in gp120 and ~4 in gp41 [8]; influenza hemagglutinin (HA) molecules have 5 to 11 PNGSs depending on subtypes, with the majority of sites residing in the globular head of the molecule [9]; hepatitis C virus (HCV) E1 has 4 PNGSs and E2 has 9 PNGSs [10]; and Ebola virus envelope glycoprotein (GP) has 17 PNGSs (15 in GP1 and 2 in GP2) [11]. HIV-1 Env and HCV E1 and E2 are so heavily glycosylated that ~50% of their respective molar masses are derived from *N*-linked glycans. Most of the critical sites in these viral glycoproteins are conserved during viral evolution, suggesting the important function of glycosylation in viral infections.

Glycosylation, which is one of the most common post-translational modifications in eukaryotic cells, is required for protein folding and maintaining protein structure. Viruses have taken advantage of this benefit at nearly every step of the viral life cycle [12]. *N*-glycosylation significantly promotes their folding and solubility, enhances subsequent trafficking of these viral proteins to their destinations, and ensures that they are properly processed and incorporated into virions. Nevertheless, glycosylation can have distinct effects that are both advantageous and detrimental to viral fitness. For example, if glycosylation occurs close to the glycoprotein processing sites, it may block the precursor cleavage by proteases and inhibit viral infection [13]; if glycosylation occurs adjacent to the receptor-binding site, it may enhance the binding affinity and promote viral infection [14,15]. In addition, the high density of glycans on virions may form a shield to impede antibody attack and promote immune evasion. However, these glycans can also become epitopes for stimulating neutralizing antibodies and the innate immune response, making viruses more vulnerable to immune clearance [16]. Thus, there are multiple selective pressures on viral envelope glycosylation that can influence the pattern of glycosylation in order to achieve the optimal fitness in their hosts [17].

## 3. Glycosylation and Unfolded Protein Response

Viruses are obligate intracellular parasites, and their glycoprotein biosynthesis and modification rely entirely on host cell machinery in the secretory pathway. Therefore, viral and host proteins are glycosylated in a similar manner by the same mechanism. Although glycans can be attached to polypeptide structures via several different mechanisms, asparagine *N*-linked glycosylation represents a fundamental and well characterized post-translational modification in eukaryotic organisms [18].

**Figure 1:**
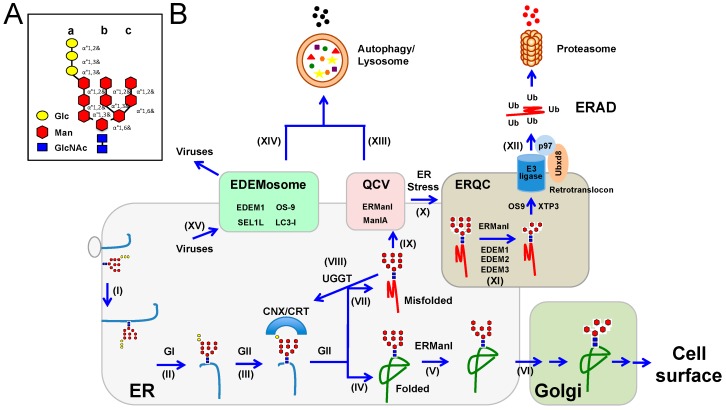
(**A**) Schematic presentation of the *N*-linked core oligosaccharide structure. The core is composed of two *N*-acetylglucosamine (GlcNAc, *blue*), nine mannose (Man, *red*), and three glucose (Glc, *yellow*) residues. a, b, and c are three oligosaccharide branches. (**B**) Schematic description of *N*-glycosylation, endoplasmic reticulum-associated protein degradation (ERAD), and endoplasmic reticulum (ER) stress pathways. Nascent polypeptides are translocated through Sec61 into the rough ER, where the core oligosaccharide is transferred from a dolichol phosphate onto asparagine residues in asparagine-X-serine/threonine (NXS/T) motifs (I). The two terminal glucose residues on the core oligosaccharide are trimmed by glucosidase I, (GI) (II), and GII (III), respectively, allowing for the association with the chaperones, membrane-bound calnexin (CNX) and and/or soluble calreticulin (CRT), which promote folding to a native conformation. Eventually, the last terminal glucose residue will be trimmed by GII, and the glycoprotein will attain a native conformation (IV), or misfold (VII). Glycoproteins that reach a native conformation will have the terminal α1,2-Man residue on the b branch removed by ER class I α-mannosidase (ERManI) (V), as a signal to allow it to traverse the canonical secretory pathway for surface presentation or secretion (VI). Polypeptides unable to reach a native conformation (VII) will engage in multiple rounds of the CNX/CRT cycle, facilitated by reglucosylation of the terminal glucose by UDP-Glc:unfolded glycoprotein glucosyltransferase (UGGT) (VIII), and trafficking between quality control vesicles (QCV) (IX) and the the ER-derived quality compartments (ERQC) (X) under ER stress. Terminally misfolded glycoproteins will be demannosylated to remove all α1,2-Man residues (XI), followed by association with lectins osteosarcoma amplified 9 **(**OS9) and XTP3-transactivated gene B protein (XTP3-B) for ERAD (XII). ERManI containing QCV are rapidly recycled through autophagy/lysosome pathways (XIII). Without interactions with client glycoproteins, EDEMosome components are degraded through an autophagy-like mechanism (XIV). Viruses can hijack EDEMosomes to form double membrane vesicles (DMVs) that serve as platforms for their replication (XV).

*N*-linked glycosylation starts from the membrane of the endoplasmic reticulum (ER), where the tetradecasaccharide precursor is assembled. This precursor consists of two *N*-acetylglucosamine (GlcNAc), nine mannose (Man, 4 are α1,2-linked), and three terminal glucose (Glc) residues distributed on three extended Man branches: a, b, and c (Glc_3_Man_9_GlcNAc_2_) (Figure 1A) [19,20]. When nascent polypeptides enter the ER lumen, the precursor is en bloc attached to Asn residues of a nascent polypeptide in a consensus Asn-X-(Ser/Thr) motif. After the attachment, these precursors are processed by a series of enzymes in both the ER and the Golgi apparatus to remold the core oligosaccharide into diverse *N*-linked glycan structures (Figure 1B). The first step in this process is the sequential removal of the two outermost Glc residues on branch A. The first Glc residue is removed by glucosidase I (GI), resulting in the di-glycosylated oligosaccharide Glc_2_Man_9_GlcNAc_2_, which is recognized by an ER transmembrane lectin malectin [21]. The second Glc residue is then removed by glucosidase II (GII), resulting in the mono-glucosylated oligosaccharide Glc_1_Man_9_GlcNAc_2_, which is recognized by two other ER lectins, the membrane-bound calnexin (CNX) and/or soluble calreticulin (CRT). Interaction with these two chaperones segregates the newly formed glycoprotein and provides access to protein disulfide isomerases (PDIs) such as ERp57, which promotes disulfide bond formation, resulting in protein folding into a native conformation. Once a protein is properly folded, GII cleaves the last Glc residue on branch A, which releases the protein from the CNX/CRT cycle. The ER class I α-mannosidase (ERManI) then cleaves the outermost Man residue on branch b on native proteins, resulting in the oligosaccharide Man_8_GlcNAc_2_. These high-Man glycans are then recognized by lectins including ER-Golgi intermediate compartment-53 (ERGIC-53), vesicular integral membrane protein of 36Kda (VIP36), and VIP36-like (VIPL), which promote trafficking from the ER to the Golgi [22]. The remaining Man residues are cleaved by the Golgi mannosidases, and the glycan remolding process is continued through the remainder of the *N*-glycosylation pathway, which generates functional glycoproteins that are delivered to the cell surface (Figure 1B).

In addition to these chaperones and enzymes that promote protein folding, the ER is also equipped with a unique quality control mechanism that extracts and degrades proteins that are not correctly folded or assembled into their native conformation, which is called ER-associated protein degradation (ERAD) [23]. In fact, the folding efficiency of glycoproteins in the ER is very low, which requires cycles of association and dissociation from CNX/CRT to ensure proper glycoprotein maturation. If glycoproteins with the Man_9_GlcNAc_2_ oligosaccharide display non-native conformations, they are reglucosylated by the UDP-Glc:unfolded glycoprotein glucosyltransferase (UGT1 or UGGT), and are subject to additional rounds of re-engagement with the CNX/CRT machinery until folding is achieved. However, if a certain time frame for the folding is exceeded, proteins may never fold properly. Misfolded proteins are sequestered into coat protein complex II (COP-II) -dependent, highly mobile ER-derived quality control vesicles (QCVs), where ERManI is enriched (Figure 1B) [24]. Because ERManI is able to excise all α1,2-Man residues when it is expressed at much higher levels in vitro [25], the enzyme may catalyze extensive demannosylation, resulting in the production of low-Man oligosaccharide Man_5_GlcNAc_2_-containing glycoprotein precursors. The removal of the a branch terminal Man residue, which is the acceptor for Glc transferred by UGGT, disables these proteins from reengagement with CNX/CRT and re-entering into the folding cycle. Importantly, the low-Man *N*-glycans represent a tag for defective glycoproteins, targeting them to ERAD [26].

With only one-tenth of the total cell volume, the ER is responsible for the synthesis of the vast majority of the secreted or membrane proteins, which account for one-third of total cellular proteins. Therefore, the ER has extremely high protein concentrations (100 mg/mL), which renders this organelle very susceptible to protein aggregation [27]. In addition, the protein folding is error prone, and this process can be further compromised by physiological and pathological perturbations. Moreover, genetic mutations may prohibit proteins from being folded properly. All these factors may cause the accumulation of unfolded or misfolded proteins. When the level of these aberrant proteins exceeds the folding and clearance capacity of the ER, known as ER homeostasis, it leads to a cellular stress response termed “ER stress”, which in turn activates the unfolded protein response (UPR) to restore the ER homeostasis [28]. ER stress is sensed by three ER transmembrane receptors: double-stranded RNA (dsRNA)-activated protein kinase (PKR)-like ER kinase (PERK), inositol-requiring enzyme 1 (IRE1), and activating transcription factor 6 (ATF6). PERK and ATF6 are in association with another ER chaperone, the binding immunoglobulin protein (BiP, or GRP78), when the cell is not under stress. BiP preferentially binds to misfolded proteins and dissociates from PERK and ATF6 under ER stress, resulting in their activation and UPR to mitigate this stress [29,30]. IRE1 is activated by the direct binding of unfolded proteins [31]. IRE1 then activates the transcription factor X-Box Binding Protein 1 (XBP-1), which in turn up-regulates ER chaperones to assist in the folding capacity of the ER as well as ERAD components to boost protein degradation. PERK phosphorylates the eukaryotic initiation factor (eIF)-2α and halts protein translation, and ATF6 up-regulates protein expression to boost the ER protein folding capacity and ERAD. However, if these objectives are not achieved within a certain time span or if the disruption is prolonged, UPR also activates pathways leading to cell death. Although PERK activation causes global inhibition of protein translation by blocking eIF-2α activity, it paradoxically enhances translation of the transcription factor ATF4. ATF4 then *trans*-activates the CCAAT/enhancer-binding protein-homologous protein (CHOP), which is a pro-apoptotic transcription factor, resulting in cell death by apoptosis [32].

## 4. ER-Associated Protein Degradation

ERAD is a protein quality control mechanism conserved in all eukaryotic cells, which is an important arm of UPR, necessary to alleviate ER stress [33]. ERAD results in the selective dislocation of unfolded and misfolded proteins from the ER to the cytosol via specific membrane machinery. ERAD targets are subsequently degraded by the cytosolic ubiquitin proteasome system (UPS) [34]. Quality control of functional proteins produced from the ER is also critical for maintenance of the ER homeostasis by eliminating unfolded and misfolded proteins. Thus, ERAD is a central element of both the secretory pathway and UPR, which targets a number of physiological and pathological substrates such as the T cell antigen receptor (TCR) [35], 3-hydroxy-3-methylglutaryl coenzyme-A (HMG-CoA) reductase (HMGCR) [35], squalene monooxygenase (SQLE) [36], Inositol 1,4,5-trisphosphate (IP_3_) receptor [37], diacylglycerol acyltransferase 2 (DGAT2) [38], heme oxygenase-1 (HO-1) [39], alpha-1 antitrypsin [35], and cystic fibrosis transmembrane regulator (CFTR) [40]. So far, more than 60 human diseases have been attributed to this pathway [41].

Although the vast majority of secreted proteins are glycosylated, the ER is responsible for the folding and assembly of both glycosylated and non-glycosylated proteins into functional complexes, which are subjected to ERAD quality control if they are misfolded. The process of ERAD can be divided into three steps: substrate recognition, retrotranslocation, and ubiquitylation/proteasomal degradation. In fact, extensive excision of α1,2-Man residues from *N*-glycans sends an important signal to trigger misfolded glycoprotein degradation, which is dependent on class I mannosidases [42].

Class I mannosidases belong to the glycoside hydrolase family 47 (GH47), which are exo-acting α1,2-mannosidases that are divided into three subfamilies [43]. The first subfamily consists of ERManI, which is supposed to cleave the outmost α1,2-Man residue on the b branch from *N*-linked glycans in the ER. The second subfamily consists of three Golgi α-mannosidase I, including GolgiManIA, GolgiManIB, and GolgiManIC, which cleave the remaining three α1,2-Man residues in the Golgi complex for *N*-glycan maturation. The third subfamily consists of the ER degradation-enhancing α-mannosidase-like proteins (EDEM) 1, 2, and 3. Although some EDEM orthologs in lower eukaryotes have detectable α1,2-mannosidase activity, such activity has not been reported for any mammalian EDEM proteins in vitro. Nevertheless, there is evidence suggesting that these EDEM proteins should have enzymatic activity in vivo [44,45]. Indeed, the extent of Man excision determines the fate of a glycoprotein, which could be either targeted to ERAD for degradation or sent to the Golgi for normal trafficking. ERManI exhibits a slow rate of enzymatic activity, which allows nascent proteins to perform multiple rounds of reglucosylation and achieve proper folding [46]. Properly folded glycoproteins should have one Man residue trimmed off from *N*-glycans by ERManI. These glycoproteins then interact with the high-Man binding lectin, ER-Golgi intermediate compartment 53 kDa protein (ERGIC-53) [47], for trafficking from the ER to the Golgi (Figure 1B). However, if glycoproteins are misfolded terminally, the remaining three α1,2-Man residues are excised from these molecules, which targets misfolded proteins for degradation [48].

It is still not completely understood how misfolded glycoproteins are subjected to such extensive demannosylation in the ER and then targeted for ERAD. Although ERManI alone may be able to complete this task, there is evidence suggesting that additional GH47 enzymes are involved. Elevation of the Golgi mannosidases has been shown to accelerate ERAD, so these enzymes may possibly be responsible for such extensive excision, likely by trafficking back to the ER via an unknown mechanism [49]. In fact, the localization of the GolgiManIA has recently been observed in QCV with other canonical ERAD machinery such as ERManI, and overexpression and knockdown can, respectively, increase or retard trimming of misfolded glycoproteins from Man_9_GlcNAc_2_ to Man_5_GlcNAc_2_ in vitro [50]. In addition, upon ER stress, QCV converge to form the ER-derived quality compartments (ERQC), where EDEM proteins are also sequestered (Figure 1B) [48]. EDEM1 and EDEM3 boost mannose trimming when overexpressed [45,51,52]. In addition, using a genomic knockout approach, it has been recently proposed that EDEM2 plays a central role in the trimming of the outmost Man residue on the b branch, whereas EDEM1 and EDEM3 should be responsible for trimming of the remaining α1,2-Man residues. Accordingly, a “double check” model for misfolded glycoproteins has been proposed, which suggests that EDEM2 catalyzes the first step of Man trimming, and EDEM1 and EDEM3 contribute the second step [44]. Under these joint actions, all four α1,2-Man residues are removed from the oligosaccharide, which is then recognized by the lectins osteosarcoma amplified 9 **(**OS9) and XTP3-transactivated gene B protein (XTP3-B) via the mannose 6-phosphate receptor homology (MRH) domain (Figure 1B) [53,54]. Misfolded proteins are targeted to specific translocation channels (retrotranslocons) for retrotranslocation in an energy-dependent manner. This process is facilitated by p97, a member of the ATPases associated with the diverse cellular activities (AAA) family, by catalyzing ATP hydrolysis [55]. The p97 ATPase is recruited by the ubiquitin-like (UBX)-domain-containing protein Ubxd8, an ER-membrane protein that plays a role in ERAD [56].

It is still mysterious how these retrotranslocons are formed and how integral membrane and lumenal ERAD substrates are exported across the ER membrane through these retrotranslocons. The first candidate channel is composed of the Sec61 complex, which is comprised of α, β, and γ subunits. The α subunit crosses the membrane 10 times, and forms a channel with the other subunits. The Sec61 heterotrimeric channel is the main translocon involved in co-translational protein transport into the ER [57]. However, there is evidence suggesting that the Sec61 translocon is also involved in retrotranslocation of ERAD substrates, implying the non-specificity and bi-directional property of this channel [58]. The second candidate is a member of the Derlin family (Derlin-1, -2, and -3) [59,60]. Derlins are integral membrane proteins that likely span the ER membrane four times and contain a rhomboid-like domain [61]. The third candidate includes the ERAD-specific E3 ligases. They have a large number of transmembrane domains, which are not only responsible for polyubiquitylation, but could act as potential exit channels for ERAD substrates [62].

In *Saccharomyces cerevisiae*, there are two major types of really interesting new gene (RING)-finger E3 ligase complexes, Hrd1 and Doa10, which mediate ERAD by targeting discrete substrates [63]. Hrd1 was the first E3 enzyme identified in the ERAD pathway during the study of HMG-CoA reductase degradation (Hrd) [64]. Hrd1 has 6 transmembrane helices in its *N*-terminal transmembrane domain and a catalytic RING domain in the soluble *C*-terminal region extended to the cytosol. Using an elegant ERAD assay reconstituted in vitro, the Hrd1-mediated formation of ubiquitin-gated protein-conducting membrane channels has been demonstrated [65,66]. Hrd1 has two mammalian orthologs named HRD1 and gp78, and its functioning E2 enzyme is known as Ubc7, which also has two mammalian orthologs, Ube2g2 and Ube2g1 [67]. Hrd1 is unstable, and must be stabilized by its co-factor Hrd3 in an equimolar ratio [68]. The mammalian ortholog of Hrd3 is SEL1L, which is required for ERAD substrate retrotranslocation [69]. Hrd1 interacts with Der1, and the Der1-Hrd1 interaction is bridged by another integral membrane protein Usa1, which is also required for Hrd1 oligomerization [70]. The Usa1 mammalian ortholog is called Herp, which also interacts with HRD1 and Derlin-1 and plays an important role in ERAD [71].

Doa10 was identified in degradation studies of Mating Type (MAT)-α2-10 (doa), which is a yeast transcription factor. Doa10 is a ~150 kDa protein that has 14 transmembrane domains, which requires both Ubc6 and Ubc7 as E2 enzymes. The Doa10 mammalian ortholog is TEB4 (MARCH6), which functions in the ERAD pathway with similar subcellular distribution and topology [72]. The Ubc6 E2 enzyme has two mammalian orthologs, Ube2j1 and Ube2j2; both are involved in ERAD [73,74].

In *Saccharomyces cerevisiae*, ERAD is executed synergistically by Hrd1 and Doa10 with minimal redundancy because they exhibit different substrate specificity. Doa10 mainly triggers ubiquitylation of specific soluble proteins and membrane proteins with degrons exposed to the cytosol; a process referred to as ERAD-C [75,76]. Hrd1 interacts with two other types of substrates, whose degradation is termed ERAD-L and ERAD-M. ERAD-L includes soluble lumenal proteins in the ER and transmembrane proteins with degrons exposed to the ER lumen; ERAD-M includes transmembrane proteins with degrons embedded into the ER membrane [63]. Simultaneous inactivation of both genes has been shown to increase the sensitivity to heavy metal-induced cellular stress and exhibit an elevated UPR.

The regulation of protein folding and the functional relation between ERAD and the UPR are much more complex in mammalian cells. In *Saccharomyces cerevisiae*, the CNX cycle does not exist due to the lack of UGGT. In addition, protein synthesis is tightly controlled at the translational level by determination of the stoichiometry to avoid surplus production, resulting in minimal dependence on the post-translational regulation of protein expression. Moreover, although the yeast ERManI ortholog Mns1p and EDEM ortholog Htm1p are indispensable for ERAD, only one EDEM ortholog is present in yeast [77,78]. Because overly active ERAD may interfere with the regular protein folding process in the ER, mammalian cells have evolved mechanisms to tightly regulate this quality control device by a combination of compartmentalization and tuning.

EDEM1 is segregated into ER-derived, LC3-I-associated vesicles, which are called EDEMosomes, where EDEM1, OS-9, and SEL1L are concentrated when they lack client glycoproteins to dislocate (Figure 1B) [79]. Notably, unlike chaperones and the other enzymes, many ERAD regulators including ERManI, EDEM1, OS-9, HERP, and SEL1L are short-lived proteins, and ERManI, EDEM1, SEL1L, and OS-9 are targeted to the lysosomal pathway for degradation [79,80]. Thus, EDEMosomes are called ERAD tuning vesicles, which deliver their content to lysosomes for disposal via an autophagy-like pathway to reduce the ERAD capacity under natural conditions [81]. Additionally, lysosomal inhibitors are able to cause the accumulation of an aggregating mutant of dysferlin in the ER when compared to the wild-type, which was used as evidence to propose that large protein aggregates are disposed of via an autophagy/lysosomal pathway, dubbed ERAD II [82]. However, under ER stress, most of these factors are highly induced, including the EDEM proteins, but not ERManI [45,83,84]. Under stress, QCV are recruited to the ERQC, resulting in the accumulation of ERManI and its glycoprotein substrates [85]. Moreover, many other ERAD components, including EDEM1, HRD1, Derlin-1, Sec61β, and Herp, are also concentrated in ERQC. Importantly, it has been found that EDEM1 stabilizes ERManI and increases its protein expression at steady-state levels [86]. Such enrichment of these critical components accelerates efficient assembly of the ERAD machinery, potentiating the degradation of misfolded glycoproteins and alleviating ER stress.

## 5. Viruses and UPR

During infection, viruses are able to hijack the host translational machinery and saturate the ER with viral proteins. Not only do viruses use the ER to generate their glycoproteins, but some even utilize the ER as their site to assemble progeny particles [87]. Such accumulation of viral proteins in the ER places a heavy demand on the protein folding machinery, which may cause ER stress, and in turn, activate the UPR, resulting in restoration of the ER homeostasis or apoptosis. So far, at least 36 viruses have been found to be able to induce ER stress, and activate the three UPR stress signaling pathways [88].

Enveloped viruses may bud through the plasma membrane or an intracellular compartment. In addition, their envelope glycoproteins are targeted to the ER for post-translational modifications and folding. Not surprisingly, many viral envelope glycoproteins are significant inducers of the UPR, which includes HCV [89], hepatitis B virus (HBV) [90], coronaviruses [91], chikungunya virus (CHIKV) [92], and retroviruses [93]. As introduced earlier, the UPR utilizes three different mechanisms to alleviate ER stress: reducing global protein translation, increasing the ER folding capacity, and enhancing ERAD by activating the PERK, ATF6, or IRE1-XBP1 pathways, respectively.

Viral infections may activate these pathways, resulting in the inhibition or enhancement of viral replication (Table 1). For example, the PERK-mediated global translation shutdown is a very effective antiviral mechanism, and a similar shutdown by PKR has been used in the interferon pathway to defend against viral infection [94]. Conceivably, viruses have evolved a number of strategies to circumvent the detrimental effect of UPR to establish productive infection. HCV is still able to produce viral proteins even when the cellular translational machinery is shut down, because these viruses have their own internal ribosome entry site (IRES) to recruit and assemble the ribosomal initiation complex for protein expression [95]. Epstein-Barr virus (EBV), herpes simplex virus (HSV), and African swine fever virus (ASFV) can counteract the PERK-mediated eIF2α phosphorylation by activating an eIF2α phosphatase PP1 [96,97,98]. In another example, the HCV E2 protein directly interacts with PERK to prevent ER stress sensing by acting as a pseudo-substrate to block PERK activity [99]. In addition to combating the UPR, viruses also take advantage of the UPR pathways to benefit their replication. For example, influenza A virus (IAV) replication is promoted by activation of the IRE1-XBP1 pathway [100]; ATF6 activation promotes ASFV, lymphocytic choriomeningitis virus (LCMV), DENV, human cytomegalovirus (HCMV), and Japan encephalitis virus (JEV) replication [101,102], and ATF4 activation enhances HIV-1 replication [103]. Thus, despite the detrimental effects, viruses have evolved to manipulate host UPR signaling pathways to promote viral infections.

Below, we will focus on the roles of ERAD played in virus replication, which is the main target of this review.

## 6. Roles of ERAD in Promotion of Virus Replication

As introduced earlier, ERAD transports unfolded/misfolded proteins from the ER into the cytosol for proteasomal degradation. Conceivably, viruses can manipulate and exploit this cellular machinery to degrade several important host factors to promote their propagation.

Herpesviruses have evolved multiple mechanisms to suppress the host immune response via ERAD. Major histocompatibility complex (MHC) molecules play an indispensable role in triggering an immediate immune response to inhibit virus infections. Herpesviruses inhibit MHC class I (MHC-I) expression by targeting these molecules to ERAD for degradation. For example, HCMV produces two transmembrane proteins, US2 and US11, and each is sufficient to bind to MHC-I heavy chains, causing their dislocation from the ER to the cytosol for degradation [108]. Notably, US2 and US11 use different mechanisms to degrade MHC-I. US2-dependent MHC-I degradation is mediated through an interaction with the E3 ligase, TRC8. This US2/TRC8 complex has been implicated in the degradation of other membrane proteins including multiple alpha-integrins, the interleukin 12 receptor (IL-12R), thrombomodulin (THBD), protein tyrosine phosphatase receptor type J (PTPRJ), and CD112 [109]. Although the signal peptide peptidase (SPP) has been shown to bind to TRC8, the US2/TRC8 complex maintains its MHC-1 degradation activity in *SPP−/−* knockout cells, suggesting that SPP binding is not related to MHC-1 degradation [110,111]. Recent reports now regard the US2/TRC8 complex as a multifunctional hub that is able to degrade a multitude of targets in order to further HCMV immune evasion [109,112]. A complex formed between US11, Derlin-1, and the E3 ligase, TMEM129, mediates MHC-I degradation via US11 [113]. Initial reports concerning US11 found an association with SEL1L and assumed that US11 mediated MHC-1 degradation could be SEL1L/HRD1 dependent. Recent literature has confirmed that while the US11/TMEM129 complex degrades MHC-1, US11 itself is degraded through a SEL1L/HRD1 axis in the absence of the client MHC-1 [113,114]. Recruitment of p97 by Ubxd8 is also crucial for US11-mediated MHC-I degradation [115]. With regard to US11, HCMV utilizes ERAD to dispose of MHC-I and its own effector protein using discrete axes for ubiquitination. Mouse gammaherpesvirus 68 (MHV68) uses another mechanism to inhibit MHC-I. MHV68 produces a protein termed MK3, which is a Ring-finger E3 ligase anchored on the ER membrane. MK3 interacts with MHC-I heavy chain molecules, and it also associates with the transporter associated with antigen processing (TAP), p97, Derlin-1, and the E2 Ube2J2. Association with Ube2J2 results in an interesting pattern of ubiquitination of non-lysine residues (the MK3/Ube2J2 complex can ubiquitinate serines as well as lysines) that leads to rapid degradation of the MHC-I by proteasomes [73,116]. Thus, herpesviruses have evolved numerous strategies to block the MHC antigen presentation and evade the host immune response to establish a persistent infection.

Primate lentiviruses also harness the ERAD pathway to promote their replication via downregulation of their receptor CD4. CD4 downregulation prevents superinfection and promotes viral release by interrupting viral receptor-envelope interactions on the plasma membrane, leading to a controlled and productive viral infection and immunodeficiency [117]. These viruses produce two accessory proteins, Nef and Vpu, to trigger CD4 degradation via two distinctive mechanisms [118]. Nef uses the endocytic pathway to redirect CD4 from the cell surface, or to interfere with the transport of newly synthesized CD4 from the trans-Golgi network (TGN) to the cell surface, resulting in CD4 dislocation to endosomes and degradation by lysosomes [119]. However, Vpu interacts with CD4 in the ER and induces CD4 proteasomal degradation via ERAD [120]. Vpu is a small transmembrane protein encoded by HIV-1 and some simian immunodeficiency virus (SIV) isolates. Vpu forms ion conductive membrane pores; it also interacts with β-transducin repeat-containing proteins (βTrCP), which are F-box/WD repeat-containing proteins that are part of the Skp1-Cul1-F-box (SCF) E3 ubiquitin ligase complex [121]. The Vpu-induced CD4 degradation is strictly dependent on the SCF-β-TrCP complex [122]. Notably, this E3 ligase complex is not associated with the ER membrane, and therefore does not normally function in ERAD. However, the degradation also requires the cytosolic ATPase p97 and its cofactors UFD1L and NPL4, which are key components of the ERAD machinery, suggesting that CD4 is degraded via ERAD [122]. Nevertheless, the degradation is not dependent on HRD1, SEL1L, and UBC7.

In addition to degradation, viruses may harness ERAD components to benefit their replication. First, ERAD can promote viral protein expression. Mouse mammary tumor virus (MMTV) is a betaretrovirus, which expresses the Rem protein in the ER. Rem has a *N*-terminal 98-amino acid signal peptide (SP), which is cleaved off by signal peptidase and retrotranslocated in a p97-dependent manner [123]. Rem SP then promotes the nuclear export of viral unspliced RNAs to the cytosol for protein expression. Similarly, hepatitis E virus (HEV) ORF2 is an *N*-linked glycoprotein, but functions as the major capsid protein. Although ORF2 is expressed in the ER, it depends on ERAD components to exit from the ER to the cytoplasm without being polyubiquitylated [124].

Second, ERAD can promote virus entry. Polyomaviruses (PyV) enter cells through the ER and then replicate in the nuclei [125]. To get from the ER to the nucleus, these viruses can cross the ER membrane into the cytosol via the ERAD retrotranslocons [126]. An example of this is mouse PyV, which uses Derlin-2, whereas simian virus 40 (SV40) uses Derlin-1 and the SEL1L complex for dislocation [126,127]. In addition, the proteasome machinery is also required for the human BK PyV exit from the ER [127].

Third, ERAD can promote virus replication. The replication of positive-strand RNA viruses normally involves the formation of double-membrane vesicles (DMVs) and convoluted membranes (CMs) by rearrangement of cellular membranes, which segregates and protects viral proteins and genomes from the host’s innate immune response. As introduced earlier, the ERAD activity can be adjusted by ERAD tuning vesicles termed EDEMosomes (Figure 1B), which display non-lipidated LC3 and segregate the ERAD factors EDEM1, OS-9, and SEL1L from the ER lumen [81]. By comparing the similarity between DMVs and EDEMosomes, it has been discovered that mouse hepatitis virus (MHV), equine arteritis virus (EAV), and JEV indeed replicate in these ERAD tuning vesicles [128]. Thus, these viruses can subvert EDEMosomes as their replication vesicles to promote infection [129].

## 7. Roles of ERAD in Inhibition of Virus Replication

Although ERAD has been frequently manipulated by a number of viruses to promote infection or attenuate immune responses, it may also function directly as an antiviral device to protect host cells from infection. Because viral envelope glycoprotein production and folding take place in the ER, these viral proteins may become the primary targets for ERAD, resulting in the inhibition of viral infection.

Primate lentiviruses, including HIV and SIV, have low levels of envelope glycoproteins on their surface, and the average copy number is ~14 Env trimers per virion [130,131]. In contrast, IFA, Sendai virus, HSV, and Moloney murine leukemia virus (MoMuLV) have much more envelope glycoproteins on their surfaces [132,133,134,135]. The exceptionally low number of Env spikes may protect HIV-1 from host immune responses [136] since almost 85% of Env proteins are retained in the ER and are degraded [137,138,139]. This degradation mechanism was not clear until we recently reported that HIV-1 Env glycoproteins are targeted for ERAD.

From completely unrelated studies, we isolated HIV-1 non-permissive (NP) and permissive (P) T cell clones N2-NP and N5-P from the original CEM.NKR human T cell line [140]. Our initial analysis uncovered that HIV-1 replication is restricted from the second round of the viral life cycle in N2-NP cells, resulting in ~1000-fold inhibition when compared to N5-P. Further transcriptome analysis by microarrays revealed that N2-NP cells overexpress the mitochondrial translocator protein (TSPO), which strongly inhibits HIV-1 Env expression [141]. TSPO interacts with the mitochondrial permeability transition pore (mPTP) complex, which includes the outer membrane protein voltage-dependent anion channel (VDAC) protein, the inner membrane protein adenine nucleotide translocase (ANT), and the mitochondrial matrix protein cyclophilin D (CypD) [142]. TSPO binds to VDAC and contributes to the regulation of the mitochondrial membrane permeability by the mPTP complex [143]. Our results suggested that TSPO overexpression could reduce the oxidative redox status in the ER, which interferes with the Env oxidative folding process, resulting in Env degradation. Consistently, the rapid Env degradation in N2-NP cells was rescued by kifunesine, an effective inhibitor of glycoside hydrolase family 47 (GH47) enzymes [144], suggesting that HIV-1 is degraded via ERAD in N2-NP cells.

To further explore the Env degradation mechanism, we investigated which of those four ER-associated GH47 enzymes was responsible for the Env degradation. Notably, when ERManI, EDEM1, EDEM2, and EDEM3 were ectopically expressed in 293T cells, only ERManI strongly inhibited Env expression in a dose-dependent manner. In addition, when the endogenous ERManI was knocked out by CRISPR/Cas9, TSPO was no longer able to suppress the Env expression [145]. These results demonstrated that ERManI should be responsible for the initiation of HIV-1 Env degradation via ERAD. Human ERManI is a 699-amino-acid, 79.5-kDa, type II membrane protein, which is divided into an *N*-terminal cytoplasmic domain (CD), transmembrane (TM) helix, lumenal ‘stem’ region, and a catalytic domain [146,147]. Using an immunoprecipitation assay, we found that HIV-1 Env interacts with the catalytic domain of ERManI [145]. The structure of this catalytic domain shows an (αα)_7_-barrel composed of 14 consecutive helices [148]. In the catalytic domain, there are seven residues that are critical for ERManI function. C527 and C556 form a highly conserved disulfide bond and were reportedly critical for protein folding [149], whereas E330, D463, and E599 were proposed as catalytic residues [148]. R334C and E397K mutations are found in nonsyndromic autosomal-recessive intellectual disability (NS-ARID) disease [150], and the R334C mutation is also found in the congenital disorders of glycosylation [151]. All these residues are required for HIV-1 Env degradation, suggesting that the mannosidase activity is important for the ERManI activity. ERManI also targets the terminally misfolded human alpha1-antitrypsin variant null (Hong Kong) (NHK) for degradation via ERAD, but neither its catalytic activity nor its catalytic domain is required for this degradation, suggesting that different mechanisms are involved in HIV-1 Env and NHK degradation [152]. We have also found that the viral protein R (Vpr) of HIV-1 enhances viral replication in monocyte-derived macrophages (MDMs) and dendritic cells (MDDCs) by rescuing Env from ERAD degradation through the ERAD (II) autophagy pathway. Compounds known to facilitate glycoprotein folding (PK11195 and As_2_O_3_) and inhibit ER α-mannosidases crucial for ERAD (Kifunensine), and those that block lysosomal proteases (Bafilomycin) rescued envelope expression and infectivity in a ΔVpr background to that of wild-type virus [153].

As aforementioned, unlike ERManI, whose expression is not responsive to UPR, the expression of the EDEMs is induced upon UPR via the IRE1/XBP activation pathway, which boosts ERAD and alleviates ER stress. Although ectopic expression of EDEMs did not inhibit HIV-1 Env expression [145], these proteins inhibit the expression of some other envelope glycoproteins. HBV expresses three surface glycoproteins, the large (L), middle (M), and small (S), which are translated from different initiation codons within the same open reading frame (ORF) and share the tetra-spanning transmembrane domains in the S protein. The *N*-terminus of the M and L protein contain additional preS2 and preS1-preS2 domains, respectively. The common S domain has an *N*-glycosylation site, and the M preS2 domain has another site. Overexpression of the surface proteins is sufficient to activate the IRE1/XBP1 pathway and elevate EDEM1, EDEM2, and EDEM3 expression. Importantly, EDEM1 overexpression destabilizes S, M, and L, and EDEM1 silencing stabilizes their expression [154]. In addition, the autophagy/lysosomal pathway, but not the proteasomal pathway, is involved in the degradation of HBV surface glycoproteins, further complicating our understanding of the viral protein degradation process via ERAD [154].

HCV has two *N*-glycosylated envelope proteins E1 and E2 on the surface of virions, which are type I transmembrane proteins expressed from a common viral polyprotein precursor. HCV infection strongly induces the activation of the IRE1 stress sensor, resulting in elevation of EDEM1, EDEM2, and EDEM3, but not the ERManI expression. Both EDEM1 and EDEM3, but not EDEM2, interact with E2, and overexpression of these two proteins induces E2 polyubiquitylation and degradation. Conversely, knockdown of EDEM1 expression or treatment with kifunesine increases E2 expression, and also reduces the interaction of EDEM1 and EDEM3 with SEL1L [155]. Taken together, these results strongly suggest that EDEM proteins are able to extract viral polypeptides from the ER quality control cycle, and degrade them via ERAD. However, since none of these proteins can target the JEV E protein to ERAD for degradation, not every viral glycoprotein is recognizable by these proteins [155]. In vivo experiments on patients with chronic liver injury were unable to identify up-regulation of UPR and ERAD elements in diseased versus control patients [156].

ERAD has also been implicated in the degradation of HCMV glycoproteins, gH and gL, via the 26S proteasome. HCMV produces at least 65 unique glycoproteins, with four homologues to the HSV glycoproteins, gH, gB, gL, and gM [157]. The glycoproteins, gH and gL, are constituents of the gcII type complexes found on the surface of HCMV virions. The gcII trimeric complex between gH, gL, and gO can initiate pH independent fusion [158]. In addition, a pentameric complex between gH, gL, and the gene products U128, U130, and U131 is able to mediate entry into different cell types via pH-dependent receptor-mediated endocytosis; a process that requires the trimeric gH/gL/gO complex [159]. Although previous studies have shown that the glycoprotein gL stabilizes the expression of gH and potentiates its surface localization [160], recent work revealed that gH is degraded via ERAD in the absence of gL [161]. Replacement of the cytoplasmic tail of gH with that of the human CD4 protein subverted gH degradation via ERAD, potentiating surface expression.

Current studies describe two paradigms for ERAD to target viral glycoproteins for degradation: ERManI-mediated, which targets HIV-1 Env, and EDEM-mediated, which can target HCV and HBV surface glycoproteins. GH47 family members share a common catalytic mannosidase homology domain of ~440-residues [52], and the three catalytic residues E330, D463, and E599 found in ERManI are all conserved in these proteins [43]. Nevertheless, there is little protein sequence homology beyond this domain among these proteins. Unlike ERManI, all three EDEMs are ER-lumenal proteins, although the signal sequence of EDEM1 is resistant to cleavage [162]. EDEM3 has two novel features including an additional protease-associated domain of unknown function and a KDEL signal for ER retention [45]. Whether or how the coordination between the EDEMs and ERManI facilitates ERAD is still a convoluted issue. Due to lysosomal degradation mediated by the *N*-terminal cytoplasmic tail, ERManI is expressed at very low basal levels in cells, and its expression is not induced by UPR [86]. Such proteolytically driven checkpoint control of ERManI expression may contribute to establish glycoprotein quality control at a baseline level, which maintains ER homeostasis without activation of IRE1/XBP1. However, if this basic mechanism fails to restore ER homeostasis, IRE1/XBP1 is induced to elevate expression of the EDEMs, which will increase ERAD. Unlike HCV and HBV, HIV-1 induces UPR, but barely activates the IRE1/XBP1 pathway, which may explain why HIV-1 Env is not directly targeted by EDEM proteins [93]. Nevertheless, these two different arms of ERAD do not exclude the role of the EDEMs in ERManI-mediated degradation. EDEMs may accelerate the release of terminally misfolded glycoproteins from the CNX/CRT cycle, and thereby help ERManI to conduct more extensive demannosylation [163]; and the association of EDEM with SEL1L may further accelerate the cytosolic delivery of misfolded proteins [164]. Moreover, EDEM1 may form a complex with ERManI, which stabilizes ERManI by the suppression of its proteolytic degradation [86]. Discrepancies concerning the localization of ERManI with various labs determining colocalization with the ER, Golgi, or ER-Golgi intermediate compartments and quality control vesicles, lends credence to both current theories that ERManI is either a Golgi checkpoint in quality control that will return misfolded proteins back to the ER for further processing, or that it resides in quality control vesicles with glycoprotein substrates as part of the CNX/CRT cycle [24,165].

## 8. Conclusions

It is well established that viruses have evolved to manipulate host UPR and ERAD to optimize their replication, whether they are ‘tuning’ host quality control to ensure the proper folding of their envelope glycoproteins, circumventing ERAD in order to prevent degradation of their viral envelope glycoproteins, or hijacking ERAD to dispose of host proteins. There are still many questions left to be answered, including the identities of the dislocons that each envelope glycoprotein is targeted to, the motifs or patterns that allow α1,2-mannosidases to differentiate between native and misfolded glycoproteins, why some viral proteins are disproportionately targeted (HCMV gH), and the roles that the UPR and ERAD play in vivo during viral infections. These exciting areas merit more extensive studies.

## Figures and Tables

**Table 1:** Viral manipulations of unfolded protein response (UPR).

Virus	UPR Pathway	Description	Ref.
HIV-1	PERK	ATF4 enhances HIV-1 replication synergistically with Tat.	[103]
IAV	IRE1	IAV infection induces IRE1. Treatment with an IRE1 inhibitor reduces viral replication. An alternate splice variant of the PB1 polymerase subunit (PB1-F2) from an avian influenza A strain has been implicated in the induction of IRE1 in chickens. ΔPB1-F2 mutant virus displayed enhanced virulence in chickens.	[100,104]
HCV	PERK	HCV E2 glycoprotein binds to PERK as a pseudo-substrate to repress PERK activation.	[99]
DENV	PERK, ATF6, IREI	PERK-mediated eIF2α phosphorylation is reversed through the viral-induced expression of GADD34, which works with PP1 to dephosphorylate eIF2α. ATF6 is activated by PERK in a cell-type specific manner. PERK and IREI knockout producer cells have decreased production of virus.	[105]
ASFV	PERK, ATF6	Ectopic DP71L expression dephosphorylates eIF2α in vitro*.* DP71L mutant viruses lack increased eIF2α phosphorylation, suggesting redundant viral factors. ATF6 activation by virus is implicated in caspase activation and early apoptosis required for viral exit.	[98,101]
EBV	PERK, IRE1, ATF6	LMP1 activates all three UPR sensors through an unknown mechanism. ATF4 is induced by the activation of PERK binding to the LMP1 promoter to stimulate LMP1 expression.	[96]
HSV1	PERK	Viral infection induces PERK and PKR, causing eIF2α phosphorylation. The HSV1 gamma(1)34.5 protein is involved in the dephosphorylation of the eIF2α through an interaction with the phosphatase PP1	[97]
CHIKV	PERK	NSP4, the viral polymerase, reduces PERK-mediated eIF2α phosphorylation.	[92]
HCMV	IRE1	HCMV late protein UL50 down-regulates IRE1 protein expression.	[106]
SARS-CoV	PERK	SARS coronavirus protein 3a activates PERK independently of IRE1 and ATF6.	[107]

HIV-1: human immunodeficiency virus type 1; IAV: influenza A virus; HCV: hepatitis C virus; DENV: dengue virus; ASFV: African swine fever virus; EBV: Epstein-Barr virus; HSV1: herpes simplex virus 1; CHIKV: chikungunya virus; HCMV: human cytomegalovirus; SARS-CoV: severe acute respiratory syndrome coronavirus; PEKR: double-stranded RNA-activated protein kinase (PKR)-like ER kinase; IRE1: inositol-requiring enzyme 1; ATF6: activating transcription factor 6; Tat: trans-activator of transcription; eIF2α: eukaryotic initiation factor-2α; PP1: protein phosphatase 1; GADD34: growth arrest and DNA damage-inducible protein 34; LMP1: latent membrane protein 1.
